# Chronic Inflammatory Diseases and Green Tea Polyphenols

**DOI:** 10.3390/nu9060561

**Published:** 2017-06-01

**Authors:** Helieh S. Oz

**Affiliations:** Department of Physiology, Internal Medicine, College of Medicine, University of Kentucky Medical Center, Lexington, KY 40536-0298, USA; hoz2@email.uky.edu

**Keywords:** chronic inflammatory diseases, green tea polyphenols, (−)-Epigallocatechin-3-gallate (EGCG)

## Abstract

Chronic inflammatory diseases affect millions of people globally and the incidence rate is on the rise. While inflammation contributes to the tissue healing process, chronic inflammation can lead to life-long debilitation and loss of tissue function and organ failure. Chronic inflammatory diseases include hepatic, gastrointestinal and neurodegenerative complications which can lead to malignancy. Despite the millennial advancements in diagnostic and therapeutic modalities, there remains no effective cure for patients who suffer from inflammatory diseases. Therefore, patients seek alternatives and complementary agents as adjunct therapies to relieve symptoms and possibly to prevent consequences of inflammation. It is well known that green tea polyphenols (GrTPs) are potent antioxidants with important roles in regulating vital signaling pathways. These comprise transcription nuclear factor-kappa B mediated I kappa B kinase complex pathways, programmed cell death pathways like caspases and B-cell lymphoma-2 and intervention with the surge of inflammatory markers like cytokines and production ofcyclooxygenase-2. This paper concisely reviews relevant investigations regarding protective effects of GrTPs and some reported adverse effects, as well as possible applications for GrTPs in the treatment of chronic and inflammatory complications.

## 1. Introduction

Chronic inflammatory diseases affect millions of people and the incidence rate is on the rise. While inflammation contributes to the tissue healing process, chronic inflammation can lead to life-long debilitation and loss of tissue function and organ failure. Tumor necrosis factor α (TNFα) is a proinflammatory cytokine that promotes various chemokines and cytokines to commence acute and chronic stages of inflammation. TNFα is released chiefly by activated macrophages, astroglia, microglia, CD4+ lymphocytes, natural killer cells (NK), and neurons [1,2,3]. TNFα release is connected with inflammation and pain related sensation in patients with inflammatory diseases like hepatitis, inflammatory bowel disease, pancreatitis, and neuropathic complications [4]. TNFα is known to contribute to the progression of neuropathic pains [5]. Soluble TNF receptors (R1 and R2) are capable of neutralizing TNFα circulation to improve pain related responses to mechanical and thermal hypersensitivity or peripheral nerve injuries [6,7]. TNFα reveals vital functions in the pathogenesis of inflammatory diseases, as inhibition of TNFα ameliorates the duration of experimental pancreatitis [2,8,9]. Genetic manipulation such as TNFα receptor 1 (TNFR1) gene deletion and anti-TNF monoclonal antibodies’ (e.g., etanercept) application improve acute inflammation in animal models [8]. Although current clinical applications to use these biological drugs may ease the inflammatory cascades and pain by reducing TNFα and other cytokines, the inflammation and pain are likely to re-surface in the patients who suffer from autoimmune diseases including arthritis and inflammatory bowel disease [7,10]. In addition, anti-TNFα monoclonal antibodies are economically unfeasible (expensive) and administration can cause potential complications [11] to provoke severe infectious diseases with viral (JC virus disease), fungal (aspergillosis) and microbial (tuberculosis) agents.

Inflammatory diseases include metabolic syndrome, nonalcoholic hepatitis, neurodegenerative diseases and gastrointestinal complications which can lead to malignancy. Indeed, the gut comprises major neuronal systems in the body and neurodegenerative disorders (e.g., Parkinson’s and Alzheimer’s) are commonly manifested with severe gastrointestinal complications. With the millennial advancements in diagnostic as well as anti-inflammatory therapeutic modalities (e.g., biological therapies, anti-TNFα monoclonal antibodies), there remains no effective cure available for those who suffer from chronic inflammatory diseases. Therefore, patients seek alternatives and complementary medications to relieve the symptoms and possibly to prevent consequences of inflammatory diseases. Yet, the safety and efficacy of these agents are not fully known as well as their possible interaction with the standard-of-care therapies. Thus, the consequences can become life-threatening, from uptake of contaminated toxic metals to complex interactions with conventional therapies [10]. 

Regular consumption of tea and plants rich in polyphenols are known for various health-promoting functions such as diuretic, anticarcinogenic, immunity-support, antimicrobial, and anti-inflammatory effects. Different relevant investigations have revealed green tea polyphenols as potent antioxidants [12,13] to play important roles in inactivation of several signaling pathways involved in inflammation. These include transcription nuclear factor-kappa B (NF-κB) mediated I kappa B kinase complex (IKK) pathways [14], TNFα [9,12,13], downregulation of cyclooxygenase (Cox)-2and B-cell lymphoma-2 (Bcl-2) activities [15], and upregulation of protective programmed cell death pathways [16,17]. Clinical trials and meta-analyses reveal constant consumption of diets rich in polyphenols to protect against chronic inflammatory diseases including cardiovascular [18] and neurodegenerative diseases [19] in humans. Consumption of a polyphenol-rich diet is linked with elevated plasma antioxidant [20], lowered markers for oxidative stress [21] and improved albuminuria in diabetic patients [22]. Additionally, polyphenols’ use includes possible production of safe plastics, nanomaterials and storage for food products. Recent studies reveal an attractive field for the possible use of these natural extracts in biopolymer formulations [23]. Polyphenols with antioxidant and antimicrobial activities are candidates for use as active compounds in bio-additives for food packaging materials to reduce the oxidation and deterioration of food [23,24,25,26] and to prevent spoilage and contamination with infectious pathogens. For instance, dibutyltin dichloride, a stabilizer used in the production of polyvinyl chloride plastics, can cause severe inflammatory complications including pancreatitis in animal models [2]. Another organic synthetic compound, bisphenol A (BPA), is utilized to produce plastics for food packaging which has the potential to disrupt endocrine hormones and to increase the risk for type 2 diabetes [27]. Polyphenolic use, as a natural packaging product, is a novel venue to replace these synthetic compounds and additives with indications of severe side effects in consumers, including elevation of chronic inflammatory complications and malignancies [2,24,25,26,27]. 

This paper reviews an insight into some of the relevant investigations regarding protective effects of green tea polyphenols and their possible applications in the treatment of chronic and inflammatory diseases. Additionally, this paper briefly discusses some of the reported side effects due to consumption of these polyphenols. 

## 2. Green Tea and Polyphenols

Leaves from *Camellia Sinensis* shrub form three types of teas including black tea, green tea and oolong tea, depending on their processing techniques. Green tea is prepared by steaming and drying the tealeaves (20% of total production). Tea extracts are commonly used as beverages, food additives or integrated into cosmetic and pharmaceutical formulations [20]. Tea extracts are rich in vitamins (B and C), minerals, polyphenols, caffeic acid, fertaric acid, tannins and volatiles. These polyphenols have been investigated for various biological and physiological activities. Tea extracts are used as coloring agents, antioxidants, and nutritional additives. Polyphenols have phenolic molecules and polymeric structures [28]. Polyphenols are ubiquitous secondary metabolites in tea and some other plants [29] and contribute to pigmentation in plant organs. Polyphenols play an important role as the mechanism of defense against environmental and biological stressors including in response to fungal and other pathogens’ attacks [16]. Polyphenols have been shown to protect against oxidative damage by inhibiting the formation of free radicals and reactive oxygen species (ROS).

**Figure 1:**
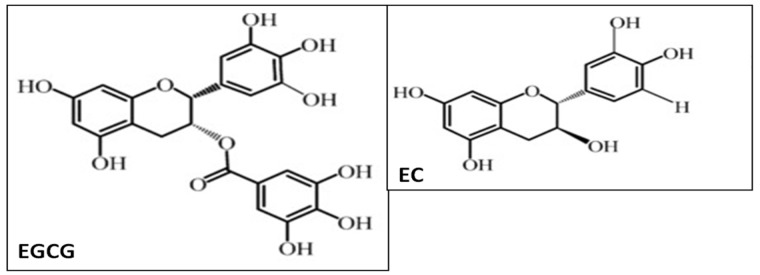
Molecular structure of (−)-epigallocatechin-3-gallate (EGCG) and (−)-epicatechin (EC).

The purified green tea polyphenols contain >95% polyphenols when analyzed with high-performance liquid chromatography (HPLC). Pure GrTP extracts contain the following percentage composition of polyphenols (each catechin): (−)-epicatechin (EC) 35%, (−)-epigallocatechin (EGC) 15%, (−)-epicatechin-gallate (ECG) 4%, and (−)-epigallocatechin-3-gallate (EGCG) 38–40% [9]. The molecular structure of EGCG and EC, two of the most abundant GrTPs, are presented in the Figure 1.

The most prevalent individual polyphenolic constituent, EGCG (98% purity), is believed to account for several therapeutic effects of polyphenols. For instance, GrTPs are reported to attenuate inflammation in different inflammatory bowel disease models [9,13,17,30,31]. Following ingestion, GrTPs are extensively dispersed amongst the organs, including the hepatic system [32]. However, the anti-inflammatory effects of GrTPs are not limited to the scavenging of toxic oxidants, as GrTPs, specifically EGCG, can block the activation of the NF-κB and the release of proinflammatory TNFα in intestinal epithelia [14]. The ability of GrTPs to inhibit NF-κB activation and release of TNFα can be responsible for the anti-inflammatory effects of tea consumption.

## 3. Inflammatory Bowel Disease and Green Tea Polyphenols

Crohn’s disease and ulcerative colitis are known chronic idiopathic inflammatory bowel diseases (IBD) mediated by immune dysregulation. In a normal gut, the number of intestinal epithelial cells (IEC) is tightly regulated to cover the surface of villi and crypts. IEC are generated by stem cells in the crypts which differentiate and migrate to the tip of villi then are “sloughed off” in approximately two to three days [15,33,34,35] and replaced with brand new epithelial cells. Therefore, the ratio of villus height in crypt stays in a constant state as it is regulated by the apoptosis (death) pathways [17]. Defective apoptosis impairs intestinal epithelial barrier function, activates immune system and macrophages, increases production of proinflammatory cytokines like TNFα and leads to IBD [17,35,36]. In Crohn’s patients, the lamina propria lymphocytes (LPL) in intestinal mucosa are chronically activated [35] with increased expression of anti-apoptotic molecules [37]. Dysregulated apoptosis in IEC and activated LPL are key pathognomonic mechanisms for IBD. Further, ROS are increased in IBD patients and implicated as mediators of intestinal inflammation [9,35].

Despite available targeted therapies and advancements in the humanized monoclonal antibodies and complementary and alternative agents [9,35], the consequences are not yet fully explored. Sulfasalazine is a standard of therapy and commonly used in IBD patients. Yet, it has severe adverse effects, such as pulmonary fibrosis, infertility, and lack of response, which ultimately leads to intestinal resection in these patients. In a bold animal study, green tea polyphenols (GrTP, EGCG) were compared to sulfasalazine for their anti-inflammatory properties. Wild type mice were given dextran sodium sulfate (DSS) for a chemically induced ulcerative colitis model [13]. Interleukin-10 (IL-10) deficient mice spontaneously develop IBD when exposed to the normal gut microbiota from their control wild type background to provoke enterocolitis similar to Crohn’s disease. Colitis and enterocolitis animals tolerated treatments with GrTP, EGCG, or sulfasalazine which was added into the diets. Treated animals similarly developed less severe symptoms compared to the sham-treated animals. The inflammatory markers (TNFα, IL-6, serum amyloid A) were significantly upregulated along with pathological symptoms but drastically decreased with GrTP, EGCG or sulfasalazine treatment. While hepatic and colonic antioxidants (glutathione, cysteine) are depleted in IBD patients and colitic models [9,13], GrTP and EGCG significantly restored antioxidant concentrations and attenuated colitis symptoms similar to sulfasalazine administration [9]. In addition, GrTP decreased disease activity and inhibited inflammatory responses in interleukin-2-deficient (IL-2^−/−^) mouse models for chronic inflammatory disease [31]. Colonic explants and LPL cultures from GrTP-treated mice had decreased spontaneous interferon-gamma and TNFα secretions [31]. In another study, lymphocytes from IBD patients and healthy subjects were chemically damaged (hydrogen peroxide) in vitro and then treated with epicatechin (0–0.1 mg/mL). A significant reduction in induced-DNA damage was discovered in lymphocytes from patients (48.6%) and normal controls (35.2%) when compared with lymphocytes from untreated subjects (both *p* < 0.001). Therefore, epicatechin significantly decreased oxidative stress in lymphocytes and supported beneficial effects of epicatechin inclusion in diets for IBD patients [38].

IKK mediates activation of NF-κB cascade in inflammatory responses to release the proinflammatory cytokine, TNFα. In vitro studies using GrTP and EGCG have shown a series of anti-inflammatory activities by inhibiting NF-κB through inactivation of I-κB kinase complex in intestinal epithelia cells. Pretreatment of intestinal cells with GrTPs (0.4 mg/mL) diminished TNFα-induced IKK and NF-κB activity [14]. The gallate group from polyphenols was required to block TNFα initiated IKK activation. When intestinal epithelial cells were transiently transfected with NF-κB-inducing kinase (NIK) for continued IKK activation, EGCG significantly decreased IKK activity in these NIK transfected epithelia [14]. Therefore, GrTP and specifically EGCG, but not other polyphenols (EC, EGC, ECG), were reported as effective inhibitors of IKK activity and as natural anti-inflammatory agents [14]. In a case-control study of 678 ulcerative colitis patients (2008–2013), increased risk for colitis was associated with serial factors as follow: (1) irregular meal times (OR: 2.287; 95% CI: 1.494–3.825); (2) consumption of fried (OR: 1.920; 95% CI: 1.253–3.254); (3) salty (OR: 1.465; 95% CI: 1.046–2.726) and frozen dinners (OR: 1.868; 95% CI: 1.392–2.854); (4) intestinal infectious diseases (1–2/year, OR: 1.836; 95% CI: 1.182–2.641); (5) frequent use of drugs such as antibiotics and NSAIDs (OR: 2.893; 95% CI: 1.619–5.312); (6) and high work stressors (OR: 1.732; 95% CI: 1.142–2.628, *p* < 0.05). In contrast, drinking tea (OR: 0.338, 95% CI: 0.275–0.488) and physical activities (1–2/week, OR: 0.655, 95% CI: 0.391–0.788; ≥3 times/week, OR: 0.461, 95% CI: 0.319–0.672, *p* < 0.05) were linked with significant protective effects in these patients [39]. These studies support possible beneficial effects of GrTP inclusion in diets for IBD patients.

## 4. Gastrointestinal Associated Malignancies and Green Tea Polyphenols

Cancer is one of the most prevalent causes of morbidity and mortality. Additionally, cancer patients are at risk for developing severe complications including diarrhea, nausea and abdominal pain during chemotherapy, mainly due to cytotoxic effects of anticancer drugs [17]. Studies suggest a protective effect of tea consumption on malignancies including those with gastrointestinal involvement mainly based on animal trials and in vitro studies. Yet, strong clinical trials and investigations are lacking to support the anticancer effects. GrTP is shown to inhibit carcinogen-induced gastrointestinal tumors in rodents [40,41,42,43,44] and in abnormal cell growth to induce apoptosis in various carcinoma cell lines [17,45,46]. GrTP and EGCG were proven to regulate apoptosis in the intestinal epithelia. In a normal gut, highly organized epithelial cells cover surfaces of villi and crypts. The number of IEC which blanket the normal gut is tightly regulated by programmed cell death (apoptosis) pathways. Dysregulated and defective apoptosis lead to IEC overgrowth and severe consequences including malignancy. Apoptosis “programmed cell death” is a process of self-destruction that can be initiated via extrinsic and intrinsic pathways. In the gut, extrinsic pathway leads to Fas-associated death domain proteins (FADD) and enzymatic activities of cysteine-aspartate specific proteases “caspasescascades” [47]. When IEC were treated with GrTP (0.4–0.8 mg/mL), they induced DNA fragmentation in a dose responsive fashion [17]. In higher concentrations (>0.8 mg/mL), GrTP caused a mixture of cytolysis and apoptosis. In addition, epithelial cells exposed to GrTP and EGCG, but not other polyphenols (i.e., EC, EGC), had increased caspase-3, caspase-8 and caspase-9 activities; but caspase inhibitors could rescue cells from imminent apoptosis [17]. Furthermore, GrTP caused activation of Fas-associated proteins with FADD recruitment to Fas/CD95 domains. Indeed, GrTP blocked NF-κB activation, yet NF-κB inhibitor (MG132) only promoted cytolysis and not apoptosis. GrTP- and EGCG-induced apoptosis in intestinal epithelia and the activation of death pathways mediated by the caspase-8 through FADD dependent pathways [17] are presenting promising results as possible anticancer agents. In a meta-analysis of 6123 gastric cancer cases and 134,006 controls, green tea consumption had a minor inverse association with risk of gastric cancer (OR = 0.68, 95% CI = 0.49–0.92). A consumption of up to five cups per day of green tea was reported safe (OR = 0.99, 95% CI = 0.78–1.27) and to prevent gastric cancer [48]. A recent case-control survey trial studied the risk factor for gastric cancer and tea consumption. Similarly, this investigation indicated protective effects of regular tea consumption (OR 0.72; CI 95%) and in a large amount (≥35 g/week) (OR 0.53; CI 95%) against gastric malignancy [49]. Future investigations may reveal use for GrTPs and EGCG as adjuvant therapies in malignancies.

## 5. Hepatic Complications and Green Tea Polyphenols

Acetaminophen [*N*-acetyl-p-aminophenol (APAP)] has been widely used as an over the counter anti-pyretic and analgesic drug since 1955. APAP overdose is a common cause of acute hepatic failure and mortality. APAP overdose is indicated in 50% of acute hepatic failures and approximately 20% of the liver transplant cases in the USA [50]. APAP-induced liver toxicity acts through many factors, including generation of ROS, glutathione (GSH) depletion, upregulation of apoptosis and Cox-2 generation, and inflammatory cytokines production. Generation of ROS causes tissue damage. GSH is a tripeptide and a powerful source of endogenous antioxidants which counteracts against the destructive deposits of free radicals. Yet, GSH sources in liver become depleted due to APAP to cause hepatotoxicity and inflammatory complications.

Current antidote practice is the use of the antioxidant Nacetylcystiene (NAC), which may not be always effective [50]. Therefore, more efficient compounds are urgently needed to protect against hepatic failure, death or liver transplants. In an investigation, mice were given a toxic dose of APAP (0.75 mg/g) by oral gavage. Animals developed profound up regulation of inflammatory markers, TNFα and Serum Amyloid A (SAA) release, as well as Cox-2 activities and Bcl-2 production. The inflammatory markers caused extensive centrilobular apoptosis, necrosis, and severe infiltration of leukocytes accompanied with generation of ROS and depletion of hepatic GSH concentration. GrTP supplementation in the diet prior to APAP injection significantly improved concentration of hepatic GSH, attenuated inflammatory markers, liver lesions and down regulated Cox-2 and Bcl-2 expression. In addition, GrTP normalized pathologically elevated hepatic enzyme activity of alanine aminotransferase (ALT) released by damaged hepatocytes, and protected against liver injury. Therefore, GrTP attenuated hepatotoxicity through normalizing antioxidants, inflammatory markers and Cox-2 and Bcl-2 activation [12,15], suggesting a potential for GrTP additives protecting against APAP toxicity.

As unhealthy diet and inactivity in urban areas are on the rise in recent decades, as are the health consequences including metabolic syndrome, the obesity epidemic and fatty liver inflammation to trigger further unforeseen socio-economic burdens. Looking for a magic wand would be a simple, effective, safe and feasible dietary compound to alleviate these public health loads. EGCG has been reported to show putative health effects including protection against inflammation and obesity. In a dose dependent study, inclusion of high dose (1.3 mg/g) EGCG in daily diets caused weight loss in DSS-treated BALB/c mice [9]. Recently, another study used very high doses of EGCG (3.2 mg/g) for three days in the same model (DSS-treated mice) and reported decreased colonic lipid peroxides and gut permeability and enhanced body weight loss [51]. Therefore, high dose EGCG might have different effects, including being responsible for lowering digestion of consumed protein and lipid and its possible application in obesity and weight loss patients. In addition, very high doses of EGCG require further safety trials.

Nonalcoholic fatty liver disease (NASH) is manifested with obesity and other complications with severe life threatening consequences. EGCG (0.05 mg/g/day) oral gavage regulated hepatic mitochondrial respiratory cascades and improved lipid metabolism, and insulin sensitivity in obese mice [52]. EGCG increased energy expenditure, and prevented oxidation of lipid substrates-stimulated by mitochondria and hepatic steatosis in this obesity model. EGCG is reported to specifically inhibit activated hepatic stellate cells by upregulating de novo biosynthesis of GSH [53]. Further, GrTP is reported to protect against NASH by decreasing hepatic steatosis and NF-κB activation in a model on a high fat diet given for eight weeks [54]. GrTP attenuated prostaglandin E2 (PGE2) accumulation and lipid peroxidation to reduce Cox-2 activity which was independent of arachidonic acid. GrTP protected against hepatic damage induced by a high fat diet in obese rats [54]. GrTP (10–20 mg/g) normalized liver malondialdehyde without affecting cytochrome P450 2E1 mRNA expression, and decreased upregulated hepatic Cox-2 activity and PGE2 elevated levels provoked by the high fat diet. In addition, GrTP attenuated increases in total hepatic short chain fatty acids without affecting the n-6/n-3 ratio and decreased total liver arachidonic acid [54]. Additionally, a double-blind, randomized clinical trial was reported in NASH patients with diagnostic ultrasonography symptoms and elevated hepatic enzymes, ALT >31 mg/dL and AST >41 mg/dL, respectively. Subjects were given green tea extract (500 mg tablet/day) or placebo for 90 days. Green tea significantly decreased hepatic enzymes; ALT and AST compared to placebo *p* < 0.001 [55]. In another randomized, double-blind trial, diabetic subjects with albuminuria received GrTP (containing 800mg of epigallocatechin-3-gallate) supplements for 12 consecutive weeks in addition to their standard of care therapy. Patients who received green tea polyphenol supplements showed significant improvements in urinary albumin-creatinine ratio (41% *p =* 0.019) compared to the placebo (standard therapy alone) group [22]. Further investigations may support the use of GrTPs in diabetic subjects and their hepatic complications including hepatotoxicity and fatty liver, as well as application in weight loss programs in obesity subjects. 

## 6. Neurodegenerative Disorders and Green Tea Polyphenols

Neurodegeneration is associated with central nervous system (CNS) disorders, such as Parkinson’s and Alzheimer’s diseases, which are caused by multiple environmental and genetic factors [56]. Neurodegenerative disorders are commonly associated with severe gastrointestinal complications, as the gut is comprised of complex neuronal systems. Alzheimer’s disease is a progressive neurodegenerative disorder characterized by amyloid β plaques formation, neurofibrillary tangles, microglial and astroglial activation leading to neuronal dysfunction and death. Microglia are primary immune cells which release proinflammatory cytokines (e.g., TNFα) and neurotoxins in the brain and contribute to neuroinflammation [1]. Neuroinflammation is a hallmark for Alzheimer’s disease. Current therapies primarily focus on symptomatic improvement of cholinergic transmission. A mechanism by which to provide neuroprotection [19] and to prevent microglial activation may be useful in the treatment of Alzheimer’s. EGCG was reported to protect neuronal cells from microglia-induced cytotoxicity and to suppress amyloid β-induced TNFα release [57]. Parkinson’s disease, the second most prevalent neurodegenerative disease, is characterized by the loss of the neurotransmitter dopamine and neuronal degeneration in the substantia nigra. Studies revealed EGCG to improve dopaminaergic degeneration and may be beneficial for Parkinson’s patients [58]. In a rat model for Parkinson’s, green tea extract or EGCG reversed pathological and behavioral modifications, demonstrating neuroprotection by decreasing rotational and increased locomotor activities. Additionally, green tea extracts and EGCG improved cognitive dysfunction by antioxidant and anti-inflammatory properties [59]. In a double blinded, randomized trial, daily consumption of 2000 mg green tea powder (containing 220 mg of catechins) for 12 months did not significantly improve cognitive function in elderly Japanese (nursing home) participants [21]. However, levels of markers for oxidative stress, malondialdehyde-modified low-density lipoprotein, were significantly lower in the green tea group (OR −1.73, 95% CI, *p* = 0.04) compared to those in the placebo arm [21].

Retinal neurodegeneration is a major cause of blindness specifically in the elderly population. Diabetic retinopathy is a recurrent complication of diabetes (type 1, type 2) which results in increased inflammation, oxidative stress, and vascular dysfunction. The inflammation and neurodegeneration may occur even before the development of clinical signs of diabetes. During the process of diabetes, the retina triggering proinflammatory signaling pathways becomes chronically activated, leading to retinal neurodegeneration and the loss of vision [61]. In a case-control clinical trial, 100 patients with diabetic retinopathy were recruited along with 100 age- and sex-matched diabetic controls without retinopathy in China. Diabetic retinopathy was confirmed from retinal photographs and the pattern of green tea consumption was collected using a face-to-face interview. The odds ratio for green tea consumption for diabetic retinopathy patients was 0.49 (95% CI: 0.26–0.90). When stratified by sex, the green tea consumption and protective effect of green tea on retinopathy was more significant in female (*p* = 0.01) than male participants (*p* = 0.63). When adjusted for age and sex, green tea consumption was reported to be significantly associated with reversed diabetic retinopathy (OR = 0.48; *p* = 0.04), high systolic blood pressure (OR = 1.02; *p* = 0.05), duration of diabetes (OR = 1.07; *p* = 0.02), and the presence of family history of diabetes (OR = 2.35; *p* = 0.04). Therefore, those diabetic patients who regularly consumed green tea (for at least one year) had a significant retinopathy risk reduction of about 50% compared with those who had not. 

EGCG with potent antioxidants is reported to neuroprotect outer retinal degeneration after sodium iodate insult [62]. Indeed, EGCG has at least twice the antioxidant potential of vitamin E or C [59]. The retinal protection with orally administered EGCG was linked with reduced expression of superoxide dismutase, GSH peroxidase, caspase-3 and suppression of 8-iso-prostaglandin generation in the retina [73], suggesting a possible therapeutic/maintenance action of EGCG in these inflammatory neurodegenerative diseases.

Stroke is a major cerebrovascular disease which results in disability and mortality, thus far with inadequate neuroprotective and neurotherapeutic agents. Tissue plasminogen activator (t-PA) is the only United States Food and Drug Administration (FDA)-approved therapy against acute ischemic stroke. Yet, clinical outcomes of t-PA depend on its short therapeutic period and grave adverse effects, such as neurotoxicity and hemorrhagic transformation. Adjuvant therapies such as EGCG may reduce the side effects and improve the outcomes [64]. EGCG has anti-angiogenic properties and a possible preventive effect against ischemic stroke via the nuclear factor erythroid 2-related factor 2 (Nrf2) signaling pathway. EGCG therapy for the acute phase of ischemic stroke has been reported to promote angiogenesis in a mouse model of transient middle cerebral artery occlusion (MCAO), conceivably by upregulating the Nrf2 signaling pathway [65]. Additionally, EGCG was shown to augment proliferation and differentiation of neural progenitor cells (NPCs) isolated from the ipsilateral subventricular zone with subsequent spontaneous recovery after ischemic stroke [64]. In a meta-analysis, 259,267 individuals were included from nine different clinical trials [65]. The amount of green tea consumption had a negative correlation with intracerebral hemorrhage and cerebral infarction. The risk increased for intracerebral hemorrhage (OR = 1.24, 95% CI: 1.03–1.49) and cerebral infarction (OR = 1.15, 95% CI: 1.01–1.30) in those who did not consume green tea, compared to those consuming more than one cup of green tea per day. The risk reduced for myocardial infarction (OR = 0.81, 95% CI: 0.67–0.98) and stroke (OR = 0.64, 95% CI: 0.47–0.86) for one to three cups of green tea consumed per day, compared to those who drank less than one cup per day. Likewise, those drinking four or more cups per day had a reduced risk of myocardial infarction (OR = 0.68, 95% CI: 0.56–0.84) compared to those who drank less than one cup per day [65]. Taken together, green tea and EGCG may exert a beneficial effect on neurogenesis, stroke recovery and prevention.

Prenatal and postnatal contact with toxic elements can cause severe consequences in newborns. Postnatal exposure of two–week-old mice pups to a single dose of valproate (0.4 mg/g subcutaneous) provokes experimental autism spectrum and related neurobehavioral abnormalities. Valproate exposed pups were treated daily with green tea extract (0.075 or 0.3 mg/g) orally for about four weeks [66]. Extensive behavioral improvements (nociceptive response, locomotion, anxiety, motor co-ordination) were detected particularly in those pups treated with 0.3 mg/g green tea extract. These modifications were consistent with reduction in oxidative stress formation as well as neuronal cytoprotection. The antioxidant prosperity of green tea polyphenols suggests a possible application in autism spectrum patients. Table 1 summarizes applied investigations into green tea and polyphenols against different chronic inflammatory diseases

## 7. Tea Polyphenols and Possible Side Effects

Tea, a popular beverage, has been consumed for many centuries. A preclinical trial described EGCG to have no detectable side effects at 800 mg/day in subjects [67]. However, some deleterious effects of tea and its GrTPs are as follows: tea is a known diuretic agent; overuse may result in dehydration. Prolonged GrTP supplementation may alter bile acid synthesis and increase hepatic oxidative stress with inflammatory hepatic injury, as reported in mice fed high cholesterol diets [60]. Weight loss may be considered a beneficial as well as a side effect of high dose GrTP (2.6 mg/g [9]) and EGCG consumption (1.3 mg/g [9], 3.2 mg/g [51]). Although tea has antimicrobial and antifungal properties, different toxic metals [68] and microbial contaminations such as *Clostridial* spp. have been isolated from unpasteurized tea [68].

*Clostridium difficle* (*C. diff*) is a facultative gram negative microbial which can cause recurrent and life threatening complications in about 0.2% of the population [69]. The recent increase in rates of recurrent *C. diff* was associated with tea consumption in the vulnerable group. A recent retrospective clinical trial in *C. diff* patients (Veteran Administration hospitals) with recurrent infection who drank tea showed the possible antimicrobial effects of tea. It was suggested that tea in the gut of these patients may reduce the normal microbiome and provoke overgrowth of the facultative pathogens [70]. However, the low number of participants in this trial requires further in-depth investigations to confirm these findings.

Gastroesophageal reflux disease (GERD) is a common chronic inflammatory disease characterized by persistent regurgitation and heartburn with increased prevalence in recent years. In a trial risk, factors were evaluated in 1685 participants. Of these, 420 (26%) suffered from GERD symptoms and the risk factors with significant effects coincided with the use of tea, coffee, smoking, NSAIDs and food indulgence [71]. The risk factors seemed to be similar to other previously reported trials, but the prevalence was remarkably higher among the studied group [71].

Finally, EGCG is counter-regulated by the presence of iron and lipocalin 2. EGCG prevents the peroxidase-catalyzed reaction by reverting the reactive peroxidase heme (compound I: oxoiron) back to its native inactive ferric state, possibly via the exchange of electrons [72]. Therefore, dietary oral intake of iron tablets can diminish EGCG, rendering it to become ineffective in inhibiting myeloperoxidase activity as an antioxidant to establish mucosal protection and anti-inflammatory effects of EGCG.

## 8. Conclusions

Chronic inflammatory diseases affect many humans worldwide, yet there is no available cure. Tea is one of the most consumed beverages globally and has been around for over 10,000 years. The polyphenols have shown varieties of possible applications, including increasing antioxidants (e.g., GSH, cysteine) depots in vital organs [9,13,35,53] and protecting against chronic inflammation in in vivo and in vitro models [31]. Studies revealed GrTPs attenuate inflammatory responses in signaling pathways, by downregulating IKK, NF-κB (0.4 mg/mL [14]), cytokines like TNFα, inflammatory markers [9,12,13,14,15,35,39], Cox-2 and Bcl-2, to protect against hepatic [12,13,14,15] and colonic [9,31,38,39] neurodegenerative complications [57,58,59,61,62,63,64,65,66,73,74] and various anti-malignancy effects [40,41,42,43,44,45,46,48,49]. Importantly, studies have revealed that GrTPs act dose dependently, as high doses (0.4–0.8 mg/mL) activate apoptotic pathways through caspases and DNA breakdown to provoke anti-malignant effects [17]. Also, GrTPs promote weight loss in high doses (EGCG 1.3mg/g/daily [9], 3.2 mg/g every three days [51]) which can be beneficial for the regulation of hepatic enzymes (10–20 mg/g [54]) and metabolism (500 mg tablet/day [55]), as well as in metabolic syndrome and obesity (EGCG 0.05 mg/g [52]); yet, this is a side effect in certain situations where weight loss is not favored. In addition, GrTPs and EGCG in very high doses (3.2 mg/g) require further safety trials. It is a common belief that constant consumption of tea provides anti-inflammatory and cardiovascular beneficial effects. Whether tea and its polyphenols provide preventive or therapeutic effects requires supporting clinical trials. In the era of antibiotic resistance and the hospital superbug epidemic, the use of GrTPs as natural antimicrobial and antifungal agents is an attractive area to be explored. However, GrTPs natural antimicrobial status that possibly alters the gut microbiome may be perceived as an adverse effect with can support facultative *C. diff* in certain vulnerable populations [70]. These warnings against GrTP utilization requires further in-depth trials before any recommendations can be implemented. Finally, GrTP has an attractive potential for use as a natural packaging material [23,24,25,26] to replace synthetic compounds with possible severe side effects by promoting chronic inflammation and malignancies in consumers [2,24,27].

## Figures and Tables

**Table 1:** Chronic inflammatory diseases and tea. Table summarizes applied investigations into green tea and polyphenols against different chronic inflammatory diseases using in in vitro, in vivo and in human trials. EGCG: epigallocatechin-3-gallate; GrTP: green tea polyphenols; IEC: intestinal epithelial cells; WT: wildtype mice, IL^-/-^ : interleukin knockout mice; APAP: acetaminophen.

Applied Investigations	Tea Extract, GrTP, EGCG	In Vitro/Animal/Human Trial	References
**Inflammatory Bowel Disease**			
	GrTP	DSS-WT mouse model	Oz et al. [13]
	GrTP, EGCG	IL-10^-/-^ spontaneous and DSS-WT	Oz et al. [9]
	GrTP	IL-2^-/-^spontaneous	Varilek et al. [31]
	Tea consumption	Patients	Niu J et al. [39]
		in vitro (patients with lymphocytes)	Najafzadeh et al. [38]
	GrTP, EGCG, EGC, ECG	in vitro IEC	Yang et al. [14]
**GI malignancy/prevention**	Tea extract	WT-mice, rats	Ju et al. [40], Metz et al. [41]; Issa et al. [42], Ohishi et al. [43]
	GrTP, EGCG	in vitrocell lines	Oz et al. [17], Isemura et al. [44], Wu [45], Basu et al. [46]
	Tea consumption	Human subjects	Zhou et al. [48]
**Hepatic complications**	GrTP	WT-mice and APAP toxicity	Oz et al. [12,15]
NASH	GrTP	Rat model	Chung et al. [54]
Diabetic	Tea extract	Patients	Borges et al. [22]
Metabolicweight loss,	EGCG	WT-mice	Oz et al. [9], Bitzer et al. [51], Santamarina et al. [52]
Fatty liver disease		WT-mice	Hirsch et al. [60]
**Neurodegenerative Disorders**			
Alzheimer‘s.	EGCG	in vitro neuronal cells	Cheng-Chung et al. [57]
Parkinson‘s disease	EGCG	Patients	Renaud et al. [58]
	EGCG	Rat model	Bitu et al. [59]
Cognitive function	Tea extract	Elderly	Ide et al. [21]
Diabetic retinopathy	Green tea	Human subjects	Ma et al. [61]
Retinalneurodegeneration	EGCG	Tat retina	Yang et al. [62]
Stroke	EGCG	WT-mice	Bai et al. [63], Zhang et al. [64]
	Tea consumption	Human subjects	Pang et al. [65]
Autism spectrum	Tea extract	WT-mice pups	Banji D et al. [66]
**Tea and Side effects**			
	EGCG	Human subjects	Chow et al. [67]
Weight loss	GrTP, EGCG	WT-mice	Oz et al. [9], Bitzer et al. [51]
Microbia, toxic metal contaminant	Tea		Ting et al. [68]
Microbial contaminant/ provocation	Tea consumption	Human subjects	Lessa et al. [69], Evans et al. [70]
Gastroesophageal reflux disease	Tea consumption	Human subjects	Vossoughinia et al. [71]
Iron deficiency	EGCG	WT-mice	Yeoh et al. [72]
